# Improving Radar Rainfall Estimations with Scaled Raindrop Size Spectra in Mei-Yu Frontal Rainstorms

**DOI:** 10.3390/s20185257

**Published:** 2020-09-14

**Authors:** Hepeng Zheng, Zuhang Wu, Lifeng Zhang, Yanqiong Xie, Hengchi Lei

**Affiliations:** 1 College of Meteorology and Oceanography, National University of Defense Technology, Nanjing 211101, China; hepeng_z@126.com (H.Z.); zhanglif_qxxy@sina.cn (L.Z.); xie_yanqiong@163.com (Y.X.); 2 Institute of Atmospheric Physics, Chinese Academy of Sciences, Beijing 100029, China; leihengchi@sina.com

**Keywords:** radar rainfall estimation, *Z–R* relation, Mei-Yu front, DSD sensor, polarimetric radar

## Abstract

Hydrological calibration of raw weather radar rainfall estimation relies on in situ rainfall measurements. Raindrop size distribution (DSD) was collected during three typical Mei-Yu rainstorms in July 2014 using three particle size velocity (Parsivel) DSD sensors along the Mei-Yu front in Nanjing, Chuzhou, and the western Pacific, respectively. To improve the radar precipitation estimation in different parts of the Mei-Yu front, a scaling method was adopted to formulate the DSD model and further derive the *Z*–*R* relations. The results suggest a distinct variation of DSDs in different parts of the Mei-Yu front. Compared with statistical radar *Z*–A*R*^b^ relations obtained by mathematical fitting techniques, the use of a DSD model fitting based on a scaling law formulation theoretically shows a significant improvement in both stratiform (33.9%) and convective (2.8%) rainfall estimations of the Mei-Yu frontal system, which indicates that using a scaling law can better reflect the DSD variations in different parts of the Mei-Yu front. Polarimetric radar has indisputable advantages with multiparameter detection ability. Several dual-polarization radar estimators are also established by DSD sensor data, and the *R*(*Z_H_*, *Z_DR_*) estimator is proven to be more accurate than traditional *Z–R* relations in Mei-Yu frontal rainfall, with potential applications for operational C-band polarimetric radar.

## 1. Introduction

Changes in the spatial and temporal patterns of climate variables associated with global warming will have an influence on regional- and catchment-scale hydrological processes [1]. According to the Global Climate Observing System (GCOS), precipitation is getting more severe with drastic changes in most of the cities worldwide [2]. For instance, Hoerling et al. [3] found an increase in both frequency and intensity of heavy rain since 1979 in the northeastern United States. Cui et al. [4] revealed the interannual variability of heavy rain in central and southern China due to the variation of large-scale environmental conditions. Investigating the response of regional rainfall events, especially extreme precipitation, will have significant implications on climate prediction. Over the last few decades, weather radars have been principally used to collect rainfall variability because of their good areal coverage as well as high-resolution measurements both in time and space [5].

Various radar quantitative precipitation estimation (QPE) algorithms, including *Z*–*R* relations (*Z* = *AR^b^*, where *Z* is the radar reflectivity factor and *R* is the rain rate), as well as polarimetric radar algorithms highly depend on surface-based DSD measurements [6,7,8,9,10,11]. Both *Z* and *R* are functions of raindrop size distributions, which can be directly derived from given DSD samples. Earlier studies tend to obtain the parameters [*A*, *b*] in *Z*–*R* relations via different mathematical fitting techniques, but the statistical results sometimes cause severe deviations [12,13]. Other scholars establish *Z–R* relations by relating coefficients [*A*, *b*] to DSD fitting models based on a scaling law formulation [14,15]. Unlike other DSD models (e.g., exponential distributions [16] and gamma distributions [17]), the scaling law allows the *Z*–*R* relation to be established without any DSD shapes imposed a priori [18]. More importantly, it provides valuable information about the intrinsic microphysical properties of the drop size distribution and its relations with radar parameters [*A*, *b*], which is likely to help improve radar rainfall estimations.

By using the scaling theory, Hazenberg et al. [19] studied the DSD characteristics and radar retrieval variability in mesoscale convective systems (MCSs), squall lines, and midlatitude cyclones over the Mediterranean region. However, no research has specifically examined the use of scaling law formulas in the Mei-Yu frontal system, a quasi-stationary front stretching over thousands of kilometers, extending eastward from southwestern China to the western Pacific through the Japanese archipelago [20,21], which is one of the most significant rainfall systems affecting the hydrological cycle in the East Asian monsoon region. Rainstorms associated with Mei-Yu (also called “Baiu” in Japan and “Changma” in South Korea) are a crucial summertime water resource and sometimes result in severe natural disasters in East Asian countries [22,23,24,25,26,27]. Meanwhile, this frontal rainstorm also intimidates the security of naval ships on the sea. Therefore, there is an urgent need to improve the performance of radar rainfall estimations within this weather system for flood monitoring, urban waterlogging prediction, and ocean storm monitoring. Based on the scaling method, one specific goal herein is to improve radar rainfall retrievals in different parts of the Mei-Yu frontal system by using in situ DSD sensor measurement data collected during three Mei-Yu rainstorms in the summer of 2014.

Following this introduction, a brief description of data and methods is presented in Section 2, the DSD properties among different parts of the Mei-Yu front are analyzed in Section 3, and radar rainfall retrievals based on DSD sensor measurements are further discussed in Section 4. Finally, Section 5 presents a summary and conclusions.

## 2. Data and Methods

### 2.1. Observational Sites and Instruments

**Figure 1:**
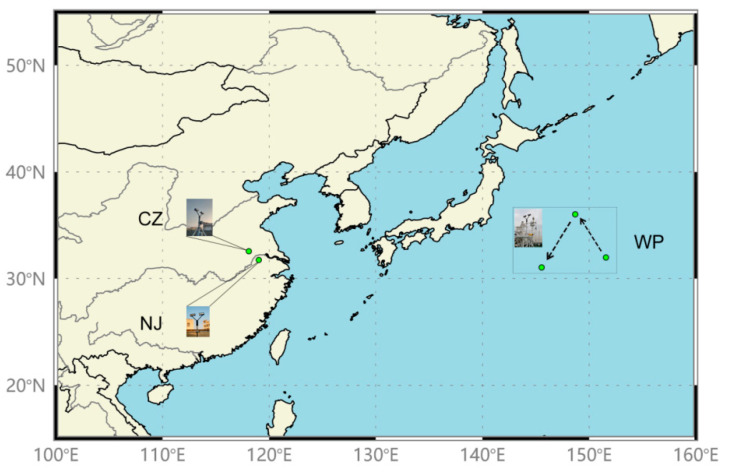
Locations of three Parsivel^2^ raindrop size distribution (DSD) sensors employed during the Mei-Yu frontal rain events. The western Pacific (WP) site is located in the southeast ocean area of Japan Sea, while Nanjing (NJ) and Chuzhou (CZ) are located in the East China continent.

**Figure 2:**
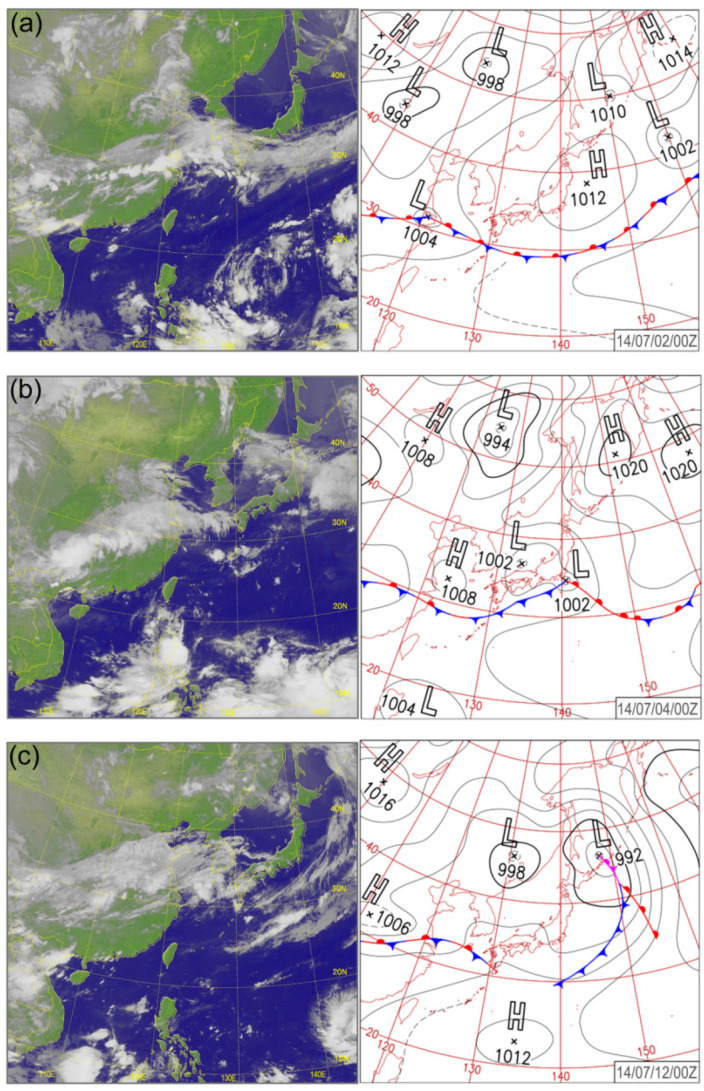
Himawari satellite image and Japan Meteorological Agency (JMA) surface weather chart at (**a**) 00 UTC, 2 July (**b**) 00 UTC, 4 July and (**c**) 00 UTC, 12 July 2014.

The data collected for the analyses consisted of three-time series of 1-min DSD data that were measured by Parsivel^2^ DSD sensors at Nanjing (NJ, 118.5° E, 32.0° N), Chuzhou (CZ, 118.2° E, 32.3° N), and the western Pacific (WP, 148.0° E, 32.1° N). DSD data in WP were measured by an onboard Parsivel^2^ DSD sensor during a marine survey over the western Pacific. Three typical Mei-Yu front rain events were simultaneously detected by the above DSD sensors on the 2nd, 4th, and 12th of July, 2014, respectively when the ship sailed across the area shown in Figure 1. Meanwhile, the satellite image from an infrared-channel sensor on the Japanese geostationary meteorological satellite Himawari-7 clearly presents a large-scale cloud belt covering southwestern China all the way to the central Pacific (Figure 2a). The rainfall is caused by a quasi-stationary front and the presence of strong low pressure as presented on the surface weather chart (Figure 2b) provided by the Japan Meteorological Agency (JMA).

The Parsivel^2^ DSD sensor [28], manufactured by OTT Hydromet, Germany, is a 2nd generation optical sensor with a near-infrared (650 nm) rectangular single-beam that is 30 mm wide, 180 mm long (for a sensor horizontal area of 54 cm^2^), and 10 mm high. It archives equivalent drop diameters sorted unevenly into 32 diameter classes from 0 to 26 mm and 32 fall speed classes from 0 to 22.4 m s^−1^. The time resolution can be selected and was set to 1 min in the present study. Nevertheless, some measurement errors, such as strong winds, margin fallers, and splashing effects are found to influence the reliable utilization of DSD sensors. Following Wu et al. [29], we implemented a procedure on data quality-control by eliminating particles with diameters larger than 8 mm or falling speeds 50% faster or slower than the empirical raindrop speed-diameter relationship proposed by Gunn and Kinzer [30]. Apart from that, 1-min samples with drop numbers less than 10 or a rain rate less than 0.1 mm h^−1^ were also excluded [31]. In order to overcome the measurement error from ship movements and tilts, the onboard Parsivel^2^ sensor was adjusted and fixed to stay perpendicular to the direction of ship pitch to best ensure the beam orientation parallel to the horizontal plane. Finally, there are overall 2611 1-min effective DSD samples for NJ, 2306 1-min effective DSD samples for CZ, and 653 samples for WP (see Table 1). The samples in NJ (CZ) are plenitudinous with the max rain rate reaching 88.22 (71.90) mm h^−1^ on 12 July (4th). Although the onboard DSD sensor observations in WP are over a relatively short period of time, the max rain rate obtained in WP could reach 48.64 mm h^−1^ on 12 July, and the max reflectivity factor is up to 48.22 dBZ on 2 July.

### 2.2. Scaling of Raindrop Size Distribution

The raindrop size distribution was computed from the Parsivel^2^ DSD sensor counts as below:

(1) $$N(D_{i})=\frac{1}{S_{eff}(D_{i})\cdot T\cdot \Delta D_{i}}\sum_{j=1}^{L}\frac{n_{ij}}{V_{j}}$$

where *N*(*D_i_*) (mm^−1^ m^−3^) is the number concentration of raindrops per unit volume per unit size interval for raindrop diameter *D_i_* (mm); *L* is the bin number of Parsivel^2^ measurements and is known to be 32; *n_ij_* is the number of raindrops within size bin *i* and velocity bin *j*; *T* is the sampling time (set to 60 s in this research), and *V_j_* (m s^−1^) is the fall-speed for velocity bin *j*. *S_eff_*(*D*) (mm^2^) is the effective sampling area [32] that considers partially detected drops across Parsivel’s laser sheet and is computed based on beam width and length:

(2) $$S_{eff}(D_{i})=180\times (30-\frac{D_{i}}{2})$$

The integral rainfall parameters, including the radar reflectivity factor *Z* (mm^6^ m^−3^), rain rate *R* (mm h^−1^), and total concentration of raindrops *N_t_* (mm^−3^) are directly derived from measured DSDs and fall velocity as below:

(3) $$Z=\sum_{i=1}^{L}N(D_{i})D_{i}{}^{6}\;\Delta D_{i}$$

(4) $$R=6\pi \times 10^{-4}\sum_{i=1}^{L}\sum_{j=1}^{L}V_{j}D_{i}{}^{3}N(D_{i})\Delta D_{i}$$

(5) $$N_{t}=\sum_{i=1}^{L}N(D_{i})\Delta D_{i}$$

To better characterize the measured DSD samples, Sempere Torres et al. [14,15] proposed a more general formulation for DSD parameterization, that is, to express the DSD as a scaling law (SL)

(6) $$N(D,\Psi )=\Psi^{\alpha_{}}g(\frac{D}{\Psi^{\beta_{}}})$$

where *Ψ* is a reference variable, commonly taken as the rain rate *R* due to its considerable variation in different rainfall systems (e.g., for deep convective system, the *R_max_* could reach up to 100 mm h^−1^), and this contributes to comparing the DSD properties over a wide range. Thus, Equation (6) can also be expressed as

(7) $$N(D,R)=R^{\alpha }g(\frac{D}{R^{\beta }})$$

where *α* and *β* are scaling parameters and *g(x)* is the scaled raindrop size distribution with x = *D*/*R^β^*. Introducing Equation (7) into (4) yields the so-called self-consistency-constraint [19]:

(8) $$6\pi \times 10^{-4}c\int_{0}^{\infty }x^{3+d}g(x)dx=1$$

where the terminal fall-speed is assumed to follow the power-law relationship of Atlas and Ulbrich [33]

(9) $$V(D)=cD^{d}$$

with *c* = 3.778 and *d* = 0.67. Different functional shapes have been proposed for *g(x)*. Herein both the exponential (*µ* = 0) and gamma distribution are employed, which can be expressed as

(10) $$g(x)=\kappa x^{\mu }\exp(-\Lambda x)$$

then ***ĸ*** can be obtained by introducing Equation (10) into (8)

(11) $$\kappa =\frac{\Lambda^{4.0+d+\mu }}{6\pi \times 10^{-4}c\Gamma (4.0+d+\mu )}$$

### 2.3. Establishment of Z–R Relations

The *Z*–*R* relationship can be established via the above SL equations as well as *Z* = *AR^b^*, the coefficients *A* and *b* thereby can be calculated by

(12) $$A=\kappa \frac{\Gamma (7.0+\mu )}{\Lambda^{7.0+\mu }},b=\alpha +7\beta$$

where *b* is related to *α* and *β*, and *A* is dependent on *g(x)*. Note that the SL method can naturally lead to power-law relationships between *Z* and *R*, without any functional shape imposed as a priori for *g(x)* [18].

The SL parameters *α* and *β*, as well as the parameters [*µ*, *Λ*] of *g(x)* for a given functional shape can be obtained by using the moment estimation method [17,34,35]. For a given DSD, the nth-order moment (*M_n_*) is defined as

(13) $$M_{n}=\int_{0}^{\infty }N(D)D^{n}dD=\int_{0}^{\infty }D^{n}R^{\alpha }g(D/R^{\beta })dD=\theta_{n}R^{\gamma_{n}}$$

(14) $$\theta_{n}=\int_{0}^{\infty }x^{n}g(x)dx,\gamma_{n}=\alpha +(n+1)\beta$$

Hence, to obtain the SL parameters *α* and *β*, *γ_n_* should be first obtained by fitting a power law between different integral moments *M_n_* and the rain rate *R* Equation (13), then *α* and *β* can be obtained according to Equation (14) that *γ_n_* should follow a linear relationship with the moment order (*n* + 1).

In addition, the parameters [*µ*, *Λ*] of *g(x)* can be calculated with the second, fourth, and sixth truncated moments (T246) [35]. And the mass-weighted mean diameter *D_m_* (mm) is calculated by the ratio of the fourth to the third moment of the DSD

(15) $$\eta =\frac{M_{4}^{2}}{M_{2}M_{6}}$$

(16) $$\mu =\frac{(7-11\eta )-{\left[{\left(7-11\eta \right)}^{2}-4(\eta -1)(30\eta -12)\right]}^{1/2}}{2(\eta -1)}$$

(17) $$\Lambda ={\left[\frac{(4+\mu )(3+\mu )M_{2}}{M_{4}}\right]}^{1/2}$$

(18) $$D_{m}=\frac{\sum_{i=1}^{32}N\left(D_{i}\right)D_{i}{}^{4}\Delta D_{i}}{\sum_{i=1}^{32}N\left(D_{i}\right)D_{i}{}^{3}\Delta D_{i}}=\frac{M_{4}}{M_{3}}$$

### 2.4. Variables of Polarimetric Radar

In addition to conventional weather radar measurements, a polarimetric radar is capable of measuring the differential reflectivity *Z_DR_* (dB) and the specific differential phase *K_dp_* (deg km^−1^) between two orthogonally polarized radar returns, which helps to significantly improve the radar data quality by reducing the impact of DSD variability on the accuracy of rainfall estimations [36]. These polarimetric variables are computed as below:

(19) $$Z_{h,v}=\frac{4\lambda^{4}}{\pi^{4}{\left|K_{w}\right|}^{2}}\int_{D_{\min}}^{D_{\max}}{\left|f_{hh,vv}(D)\right|}^{2}N(D)dD$$

(20) $$Z_{DR}=10\log_{10}\left(\frac{Z_{h}}{Z_{v}}\right)$$

(21) Kdp=10−3180πλRe{∫DminDmax[fh(D)−fv(D)]N(D)dD}

where *λ* is the radar wavelength; *K_w_* is the dielectric constant factor of water and *f_hh_*_,*vv*_(*D*) (*f_h_*_,*v*_(*D*)) is the backscattering (forward-scattering) amplitude at the horizontal or vertical polarizations. In this research, the assumed C-band (50 mm) dual-polarization radar variables are derived from the Parsivel^2^ DSD sensor data by the use of T-matrix scattering approach for non-spherical particles [37]. The mean and standard deviation of the canting angle is set to 0° and 7° respectively with a Gaussian model. The raindrop temperature is assumed to be 20 °C for summer season and the relation of axis ratio *r* and its equivalent diameter *D* (mm) based on Brandes et al. [38] is used as below:

(22) $$r=0.9951+0.02510D-0.03644D^{2}+0.005303D^{3}-0.0002492D^{4}$$

### 2.5. Assessment Statistics

To evaluate the overall quality of radar QPE algorithm and eliminate the influence from different rainfall totals over three sites in the meantime, the normalized absolute error (NAE), the normalized mean bias (NB), and the coefficient of determination (r^2^) are examined in each site as defined below:

(23) $$\mathrm{NAE}=\frac{\sum_{j=1}^{N}\left|R_{\mathrm{radar}}-R_{j}\right|}{\sum_{j=1}^{N}R_{j}}$$

(24) $$\mathrm{NB}=\frac{\sum_{j=1}^{N}\left(R_{\mathrm{radar}}-R_{j}\right)}{\sum_{j=1}^{N}R_{j}}$$

(25) $$r^{2}=1-\frac{\sum_{j=1}^{N}{\left(R_{\mathrm{radar}}-R_{j}\right)}^{2}}{\sum_{j=1}^{N}{\left(R_{j}-\bar{R_{j}}\right)}^{2}}$$

where *N* is the number of effective samples in rain event, *R_j_* and *R*_radar_ represent the observed rain rate by DSD sensors and the radar-retrieved rain rate for each sample, respectively. Note that the specific expression of *R*_radar_ is not given here, since the radar-retrieved results are from either conventional radar or polarimetric radar (e.g., *R*_radar_ = (*Z_j_*/*A*)*^1/b^* for conventional radar).

### 2.6. Classification Scheme of Rain Types

Based on DSD sensor measurements, the DSD samples were further classified into two fundamental rain types (convective rain and stratiform rain). Several rainfall classification schemes have been well developed [34,39,40]. Nevertheless, the results of Tokay and Short [34] were obtained from rainfall clusters in the tropics, which is inappropriate in our Mei-Yu study because of the regional variability. The categorization scheme of Testud et al. [39] was based on the variability of rain rate (*R*) with time. For a time series of the rain rate {*R_i_*}, the sample k was classified as stratiform only if the R values of ten adjacent values from *R_k_*_−5_ to *R_k +_*_5_ were all less than 10 mm h^−1^, otherwise, it was assumed to be convective. And the classification scheme of Bringi et al. [40] was based on the standard deviation value (*σ_R_*) of the rain rate R. A threshold of *σ_R_* = 1.5 mm h^−1^ was used for the classification of stratiform and convective rain. In this research, two classification schemes were adopted together to separate total samples into convective and stratiform clusters. More specifically, for ten consecutive 1-min samples, if the *R* values of ten adjacent values were all less than 10 mm h^−1^ and the standard deviation *σ_R_* was less than 1.5 mm h^−1^, then the sample was defined as stratiform, otherwise, it was classified as convective. As a result, NJ, CZ, and WP consisted of 77% (23%), 90% (10%), and 41% (59%) of stratiform (convective) rainfall DSD samples, respectively. The results indicate the presence of strong convective rain over the Pacific Ocean.

## 3. DSD Properties from Parsivel^2^

### 3.1. Analyses of Drop Size Spectra

**Figure 3:**
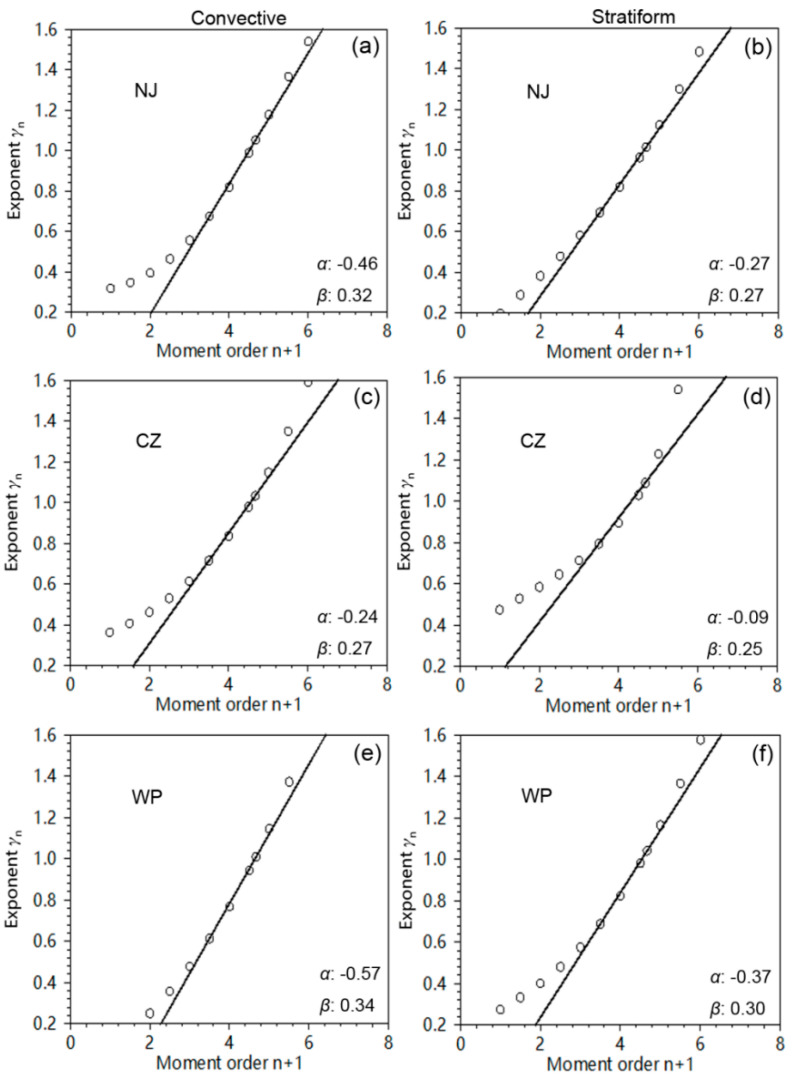
Scaling analysis of the drop size spectra for the Mei-Yu events in July 2014. The exponent *γ_n_* versus the moment order n + 1 are plotted in three different parts of the Mei-Yu front with two different rain types (left panels (**a**), (**c**), and (**e**) for convective rain, right panels (**b**), (**d**), and (**f**) for stratiform rain). Additionally shown in each panel are the scaling exponents (*α*, *β*).

**Figure 4:**
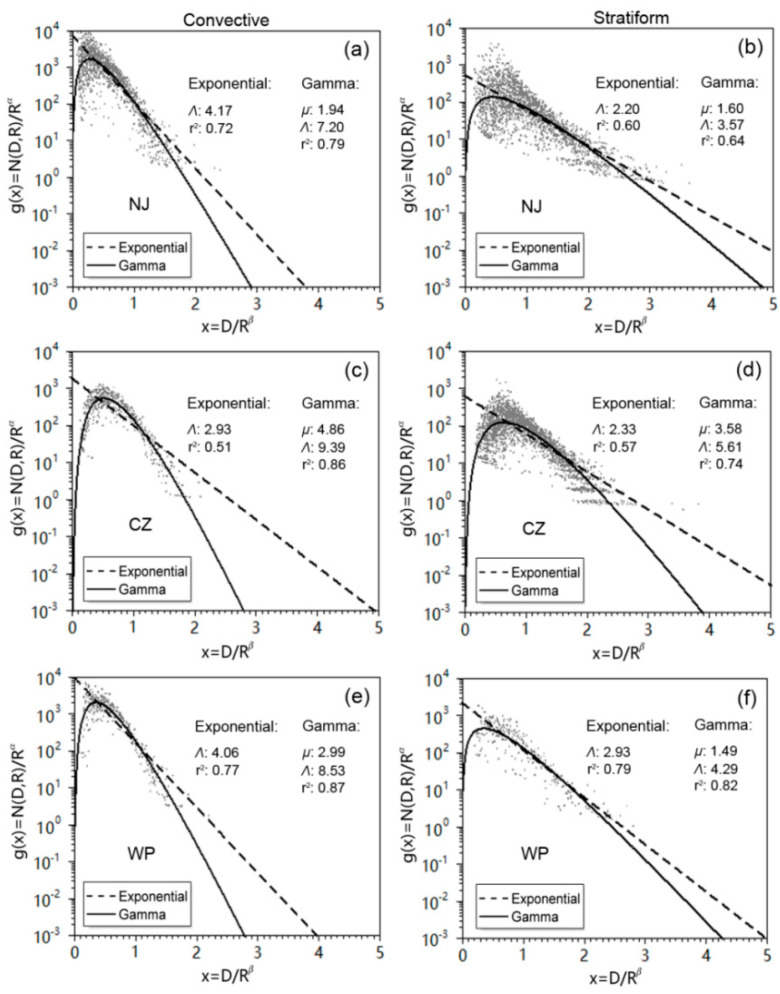
By using the moment estimation method, the exponential (dashed) and gamma (solid) shapes in three different parts of the Mei-Yu front are obtained and presented, together with the scaled drop size spectra. The coefficients of the distributions [*µ*, *Λ*] and the coefficient of determination r^2^ are presented as well. The left panels (**a**), (**c**), and (**e**) represent convective rain, while the right panels (**b**), (**d**), and (**f**) represent stratiform rain.

In Figure 3 and Figure 4 the analyses of the raindrop size spectra are presented. Figure 3a presents the values of the exponents *γ_n_* calculated by the least squares method between different integral moments *M_n_* and the rain rate *R* (Equation (13), for 0 ≤ *n* ≤ 6). The fit was performed over the central moments (1 ≤ *n* ≤ 5) in order to minimize instrumental effects that might occur in measuring the smaller and larger raindrops by Parsivel DSD sensors. Except for the lowest-order moments, the overall fit in panels (a) and (b) of Figure 3 was very good. Based on the estimated values of *γ_n_*, the coefficients *α* and *β* can be further obtained via Equation (14), and the results are also shown in each panel of Figure 3. For stratiform situations, it can be found that the sum of two coefficients was positive in CZ, negative in WP, and equal to zero in NJ, which suggests an uneven distribution of rainwater in different parts of the Mei-Yu front. Note that the coefficient *α* had a negative value under all circumstances in the Mei-Yu front. This was different from the results of squall line (*α* = 0.24) and MCS (*α* = 0.22) as reported by Hazenberg et al. [19], which suggests that the DSD characteristics in the Mei-Yu front were unique and worth our further research.

To develop deeper insight of the Mei-Yu microphysics, the scaled raindrop spectra along with two different functional shapes are presented in Figure 4. It can be seen that the normalized DSD data exhibited an exponential shape, which was well fitted by both exponential and gamma distributions, as shown by the high values of the coefficient of determination r^2^ calculated by Equation (25) and used for measuring the fitting efficiency of the relations. Comparing two rain-types, a wider normalized spectrum can be noticed in stratiform rain clusters compared to convective rain clusters, which indicates completely different DSD characteristics of the Mei-Yu front unlike other strong convective systems such as squall lines. Comparing the three regions from different parts of the Mei-Yu front, the max particle concentration of convective rain was largest in WP and smallest in CZ, and the spectra of stratiform rain were widest in NJ and narrowest in CZ. This reflects the distinct DSD variability in different parts of the Mei-Yu front. The SL method was able to provide information about the intrinsic microphysical properties of DSD. It was reported by Uijlenhoet et al. [41,42] that precipitation may reach an equilibrium state, where the collision-coalescence and collision-breakup processes of raindrops are nearly in balance, causing all characteristic raindrop sizes to remain constant when the obtained SL coefficients (*α*, *β*) satisfy *α* = 1, *β* = 0. From Figure 3d one can notice that CZ had the smallest *β* value (0.25) for stratiform rain as compared to NJ (0.27) and WP (0.30), which corresponds to its more uniform DSD and narrower width of spectra in Figure 4d. A different rainfall control mechanism, on the other hand, can be found in the stratiform rain of NJ. Note that the sum of coefficients *α* and *β* was equal to zero in NJ as presented in Figure 3b, which results in non-homogeneous precipitation [43] and distinct variations of drop size. This is also called a “size-control” mechanism.

### 3.2. Statistics of DSD Parameters

**Figure 5:**
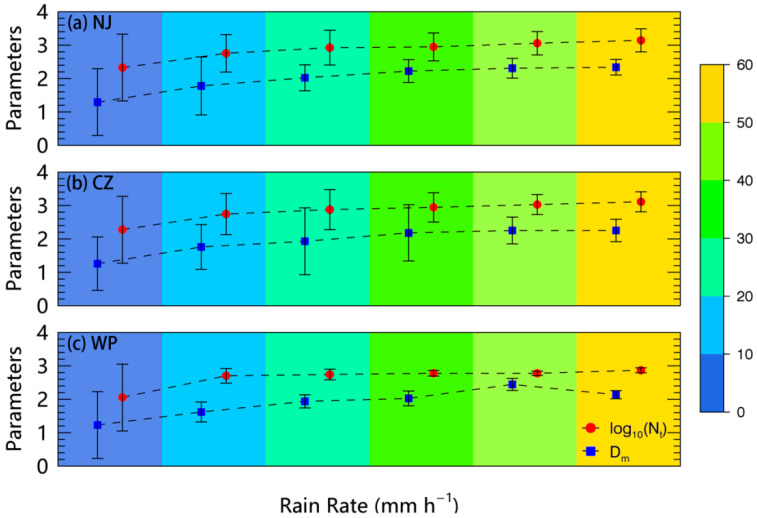
Evolution of *D_m_* (mm) and *log*_10_*(N_t_)* (m^−3^) versus rain rate (mm h^−1^) for DSD samples observed in (**a**) NJ, (**b**) CZ, and (**c**) WP. The red circles represent parameter *log*_10_*(N_t_)*, while the blue rectangles represent parameter *D_m_*. Symbols are plotted at the mean for ordinate within each rain rate class. The color bar at right side represents six classes of rain rate with an interval of 10 mm h^−1^. The maximum and minimum of two parameters are given in the whiskers of each symbol.

To further study the rainfall microphysics in different parts of Mei-Yu front, Figure 5 shows the evolution of *D_m_* and *log*_10_*(N_t_)* versus rain rate for DSD samples observed in three parts. The parameters *D_m_* (blue rectangle) and *log*_10_*(N_t_)* (red circle) are presented in six classes of rain rate with an interval of 10 mm h^−1^ and the ranges of both parameters are indicated by the whisker of each symbol in Figure 5. Additionally given in Table 2 is the number of samples in each rain rate class. Remarkably, both *D_m_* and *log*_10_*(N_t_)* increased with an increase in rain rate at the first three classes (*R <* 30 mm h^−1^). However, at the last three classes where *R >* 30 mm h^−1^, the parameters seemed to become stable with *D_m_* values staying approximately at 2.16 (2.12) mm in NJ (CZ) and 2.08 mm in WP, while the *log*_10_*(N_t_)* values still increased slowly with an increasing rain rate. Note that the ranges of both *D_m_* and *log*_10_*(N_t_)* become smaller and smaller with rain rate increases. At higher rain rate classes, the DSDs may reach an equilibrium state where coalescence and breakup processes of raindrops are in close balance [44]. Under equilibrium conditions, *D_m_* remained nearly constant, while any increase in rain rate was mainly attributed to an increase in the concentration of raindrops [8], which was also called a “number-control” mechanism. The unique characteristics of *D_m_* and *log*_10_*(N_t_)* in our study were consistent with this theory but further demonstrate that the rainfall in the Mei-Yu front is unevenly distributed with larger raindrops and more abundant particles in its continental part.

### 3.3. µ–Λ Relations

It has been widely reported that *µ* and *Λ* show rather similar behaviors and a very strong correlation [35,45]. Studies have also shown that radar rainfall estimations can be improved after adjusting the *µ*–*Λ* relationship to ground DSD sensor observations [46]. Over the past few years, it was found that *µ*–*Λ* relations vary with respect to rain types, climate regimes, and terrains [35,46,47,48]. Hence, the *µ*–*Λ* relation needs to be adapted to the Mei-Yu frontal system.

Following the method of data-processing in Zhang et al. [35], the DSDs with *R* > 5 mm h^−1^ and *N* > 1000 were fitted via the moment method to obtain a second-degree polynomial *µ*–*Λ* relation in three parts of the Mei-Yu front. The relation for the NJ is given by

*Λ* = 0.0156*μ*^2^ + 0.636*μ* + 1.533 (26)

For CZ the relation is given by:

*Λ* = 0.0072*μ*^2^ + 0.688*μ* + 1.426 (27)

For WP the relation is given by:

*Λ* = 0.0244*μ*^2^ + 0.608*μ* + 1.351 (28)

For the entire Mei-Yu front is given by:

*Λ* = 0.0080*μ*^2^ + 0.741*μ* + 1.432 (29)

**Figure 6:**
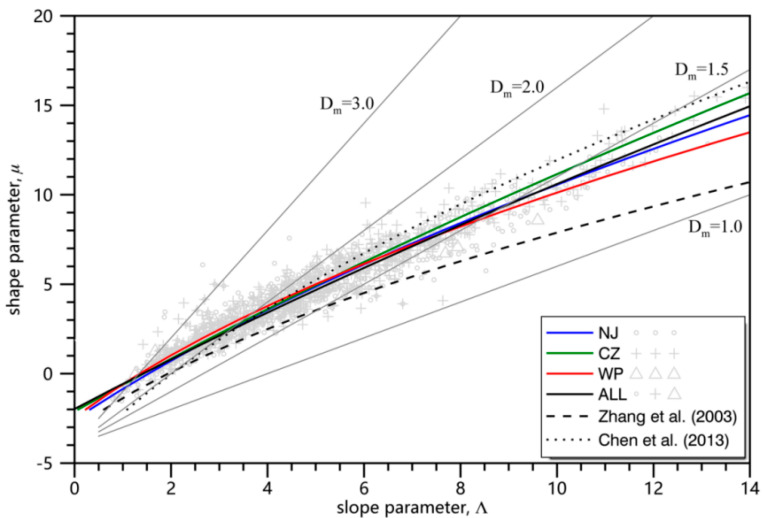
*µ–Λ* scatterplots and fitting curves based on Parsivel^2^ DSD sensor observations in three parts of the Mei-Yu front. The gray lines correspond to the relationship *ΛD_m_* = 4 + *μ* given the values of *D_m_* = 1.0, 1.5, 2.0, and 3.0 mm. The gray circles (crosses) represent precipitation in NJ (CZ), and the gray triangles represent precipitation in WP. The dashed line and dotted line represent the empirical *μ*–*Λ* relations reported by Zhang et al. [35] and Chen et al. [45], respectively.

The fitting lines and their corresponding scatter plots are presented in Figure 6. Given the same *Λ*, it’s notable that the parameter *µ* of the WP is less than that of NJ (CZ). The *μ*–*Λ* relation can also be formulated as *ΛD_m_* = 4 + *μ* [35,45]. As presented in Figure 6, more samples of precipitation in NJ (gray circle) and CZ (gray cross) lie in the larger *D_m_* region. Such characterization is consistent with the results presented in Figure 5.

Compared with the statistical results in other regions, presented together in Figure 6, the results that we derived in three parts of the Mei-Yu front were right between those obtained by Zhang et al. [35] and Chen et al. [45]. This could be attributed in part to DSD sensor uncertainty. The Parsivel^2^ sensors used in our study generally underestimate small drops [31,37,49,50], resulting in a larger *µ* value than 2DVD (two-dimensional video disdrometers) observations in Zhang et al. [35]. Using the same type of Parsivel instruments, the *μ*–*Λ* relation obtained by Chen et al. [45] overestimated the large drops and underestimated the small drops. This could be the reason that only a small area of Mei-Yu is considered in Chen et al. [45]. Thus, it is crucial to obtain the unique *µ*–*Λ* relationship for different parts of the Mei-Yu front.

## 4. QPE of Radar

### 4.1. Z–R Relations

It is reported that a single, unique *Z–R* relation does not exist, and it strongly depends on the geographical location and physical conditions of rainfall [51]. For instance, the National Weather Service’s (NWS) Weather Surveillance radar-1988 Doppler (WSR-88D) precipitation processing subsystem recommends *Z* = 250*R*^1^.^2^ [6] for tropical systems, and the Next Generation Weather radar (NEXRAD) utilizes *Z* = 3 00*R*^1^.^40^ for mid-latitudes [52]. It was also reported by Rosenfeld and Ulbrich [53] that there are significant differences in the *Z–R* relations between maritime precipitation and continental precipitation. However, owing to the particularity of geographical location, in situ observations of maritime precipitation are quite difficult. The Mei-Yu front extends from eastern China to WP. There are few comparisons to demonstrate the variability of *Z–R* relations between its continental and maritime parts. Therefore, combined with unique DSD observations, the study of *Z-–R* relations of precipitation in different parts of the Mei-Yu front is required to improve the quality of radar precipitation estimations in a specific region.

**Figure 7:**
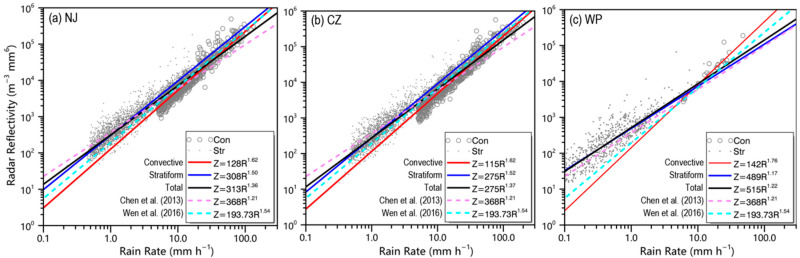
*Z-R* scatterplots and fitting curves for Mei-Yu precipitation in (**a**) NJ, (**b**) CZ, and (**c**) WP. The gray circles indicate convective precipitation samples, the gray dots indicate stratiform precipitation samples, the solid red line indicates the convective precipitation fitting curve, the solid blue line indicates the stratiform precipitation curve, the solid black line indicates the total precipitation curve, and the two dashed lines indicate the empirical relation *Z* = 368*R*^1^.^21^ for convective precipitation suggested by Chen et al. [45] and *Z* = 193.73*R*^1^.^54^ for stratiform rain obtained by Wen et al. [55].

To compare the scaling law method with the other fitting methods, Figure 7 presents the results of *Z–R* fittings using the least squares method, which is implemented by deriving the coefficient *b* from the fit and set the coefficient *A* to force the total rainfall to agree (i.e., *A* = (Σ*Z*^1/^*^b^*/Σ*R*)*^b^*), because the fitting provides greater uncertainty in estimating *A* than it does *b* [54]. Additionally presented in this plot are scatter plots of rain rate *R* versus radar reflectivity factor *Z* as measured by the DSD sensor. In addition, *Z* = 368*R*^1^.^21^ for convective precipitation observed with Parsivel sensor during Mei-Yu [45] and *Z* = 193.73*R*^1^.^54^ for stratiform rain observed with 2DVD sensor during Mei-Yu [55], are also provided in the corresponding colors for comparisons with previous research in terms of different rain types. According to the results, the *Z–R* relations of precipitation within different parts of the Mei-Yu front were significantly different. Comparing the precipitation in NJ (CZ) and WP, coefficient *A* and exponent *b* in *Z*–A*R*^b^ relations had an inverse relationship. A larger *b* value can be found for total precipitation in WP, which can be explained via Equation (12) since WP has a larger *β* (see Figure 3e,f).

Compared with *Z* = 368*R*^1^.^21^ for convective rain observed by Chen et al. [45], the relation overestimated the convective precipitation in three parts when rain rate was below 10 mm h^−1^, whereas it underestimated the convective precipitation when the rain rate was above 10 mm h^−1^. Moreover, the relation was quite similar to our *Z* = 489*R*^1^.^17^ relation for stratiform precipitation in WP, which further reflects the distinct microphysical variability within different parts of Mei-Yu precipitation. Compared with *Z* = 193.73*R*^1^.^54^ for stratiform rain observed by Wen et al. [55], the relation was very close but slightly lower to our *Z* = 308*R*^1^.^50^ (*Z* = 275*R*^1^.^52^) relationship for stratiform rain in NJ (CZ), which might be related to the different method of rain categorization used and the recognition of shallow rain type in Wen et al. [55]. Due to the lack of radar observations, shallow rainfall was recognized as stratiform rain in our surface DSD sensor-based categorization schemes. Though the influence from rain type categorization herein was not very obvious, it is worth further improvement, and we leave it for future research. Based on the above analyses, as well as previous studies [56,57,58], it is suggested that DSD variability related to the precipitation microphysics in different types of rain and terrain are major sources of the diversity of *Z–R* relations.

Besides the standard Z = 300R^1^.^4^ relation (STD) and the relations using the least-squares fitting method (LS), the optimal *Z*–*R* relations for the normalized exponential (EXP) and gamma (GAM) distributions in each site were also obtained via Equation (12). Their values are shown in Table 3. The fitting results were similar for the EXP, GAM, and LS methods and varied mainly with respect to the value of coefficient A. Compared to the LS method, both coefficients A and b were larger in EXP and GAM methods. The result of GAM, however, was closer to that of the LS method in comparison to the EXP method.

For the assessment of radar rainfall retrievals, the statistics calculated via Equations (23) and (24) are shown in Table 4. Comparing the four estimation methods at the same site, the NAE in Table 4 for the GAM method was smallest under both stratiform and convective situations, while the NAE for the STD method was rather poor as compared to the other three techniques. In comparison with statistical LS estimations, we calculated the difference of NAE errors between LS and GAM method in three sites for both stratiform rain (8.7% for NJ, 9.9% for CZ, 15.3% for WP) and convective rain (1.2% for NJ, 1.2% for CZ, 0.4% for WP), and found that the scaled gamma spectra method showed an impressive improvement in both stratiform (33.9%) and convective (2.8%) rainfall estimations of total Mei-Yu frontal rainfall. This further indicated that using scaling law fitting can well reflect the variation of DSD in different parts of the Mei-Yu front. The EXP method had a better performance in stratiform rain than convective rain, which indicated that the EXP method was very sensitive to rain rates. For scaled DSD samples, the GAM method yielded smaller NAE and NB for both rain types in comparison to the EXP method, which could be due to its more representative description of raindrop size spectra. On the other hand, the NB in Table 4 for EXP and GAM methods were both negative, while the opposite results were noticed for LS and STD methods. This indicated that the normalized spectra shapes tended to underestimate the Mei-Yu precipitation, whereas the LS method and STD relation usually overestimated the Mei-Yu rainfall.

### 4.2. Polarimetric Radar Applications

One of the sources of uncertainty in radar precipitation estimates, including *Z–R* relations, resulted from variable raindrop size distributions. It is expected that uncertainties in radar QPE due to variability in precipitation microphysics and target identification can be reduced by the use of dual-polarization techniques, which transmit both vertically and horizontally polarized electromagnetic waves. Because larger raindrops are not spherical, their backscattering cross-sections are different for the two polarizations; hence, the returned power and the Doppler shift within the two channels vary. As a result, the returned polarimetric signals, such as *Z_DR_* calculated by Equation (20) and *K_dp_* calculated by Equation (21), yield valuable information regarding hydrometeor size, shape, orientation, and microphysical phase [59]. Diverse rainfall estimators based on dual-polarization radar data have previously been reported, such as *R*(*Z_H_*, *Z_DR_*), *R*(*K_dp_*) [37,46,59,60]. Therefore, the application of polarimetric radar as new rainfall estimators may help reduce the uncertainty due to DSD variability among different parts of the Mei-Yu front.

In this study, C-band polarimetric radar parameters *Z_H_* (mm^6^ m^−3^), *Z_DR_* (dB), and *K_dp_* (deg km^−1^) were calculated following *Zhang* et al. [37] for the Brandes drop shape assumption using Parsivel^2^ sensor data observed during the Mei-Yu season (see Section 2.4). *R*(*Z_H_*, *Z_DR_*) and *R*(*K_dp_*) are usually obtained in the form of *R* = a*Z_H_*^b^*Z_DR_*^c^ and *R* = a*K_dp_*^b^ respectively. By using the least-squares method, the best-fit results derived from DSD for the *R*(*Z_H_*, *Z_DR_*) relationship is

*R* = 0.0023*Z_H_*^0^.^926^*Z_DR_*^−0^.^97^ (30)

for the *R*(*K_dp_*) relationship, the equation is fitted as

*R* = 19.2*K_dp_*^0^.^70^ (31)

The comparison results and statistics are shown in Table 5. Compared to the other two, *R*(*Z_H_*, *Z_DR_*) performed the best in the Mei-Yu front with an NB of −3.8% and an NAE of 9.2% for NJ, an NB of −4.9% and an NAE of 10.7% for CZ, and an NB of 25% and an NAE of 25.5% for WP. The results indicated that polarimetric estimation was more accurate than the *Z–R* relationship. We recommend in this work to use *R*(*Z_H_*, *Z_DR_*) for estimations of Mei-Yu rainfall. Nevertheless, *Chen* et al. [10] derived three different rainfall estimators, *R*(*Z_H_*), *R*(*Z_H_*, *Z_DR_*), and *R(K_dp_)*, from two-year 2DVD observations of Mei-Yu systems and found that *R(K_dp_)* was the most accurate among the three estimators. This is because the results of *Chen* et al. [10] were obtained only by using DSD data collected in eastern China, while the oceanic part of the entire Mei-Yu front was not considered, which further implies the microphysical variability in different parts of the Mei-Yu front is of great significance.

## 5. Summary and Conclusions

In the work, we derived scaling law DSD models in different parts of the Mei-Yu front by using the DSD samples measured from Parsivel^2^ sensors, and DSD-based relations were further derived to improve the accuracy of quantitative radar precipitation estimations. The major conclusions can be drawn as below:

(1) The spectral width of normalized DSD in NJ stratiform rain was widest among different parts of the Mei-Yu front, resulting in a “size-control” drop size distribution. The max particle concentration of convective rain was largest in WP, indicating a strong oceanic convective rainstorm. The average *D_m_* value in heavy rain was larger in NJ (2.16 mm) and CZ (2.12 mm) than in WP (2.08 mm) of the Mei-Yu front. Given the same *Λ*, the parameter *µ* of WP was less than that of NJ (CZ).
(2) The *Z–R* relations were estimated by using four methods (STD, LS, EXP, and GAM). The scaled spectra shapes (EXP and GAM) tended to underestimate the Mei-Yu precipitation, whereas the statistical LS method and STD relation usually overestimated the Mei-Yu rainfall. The EXP method had a better performance in stratiform rain than convective rain, which indicates that it is very sensitive to rain rates. The GAM method performed best compared to the other three methods in Mei-Yu rainfall estimations. In comparison with LS estimations, the GAM method showed a considerable improvement in both stratiform (33.9%) and convective (2.8%) rainfall estimations of the Mei-Yu front.
(3) Several polarimetric variables are calculated by the use of DSD sensor data, such as *Z_H_*_,*V*_ and *Z_DR_*. Furthermore, we derived empirical *R*(*Z_H_*, *Z_DR_*) and *R*(*K_dp_*) estimators to improve the polarimetric radar rainfall estimation in the Mei-Yu front and found that the *R*(*Z_H_*, *Z_DR_*) estimator demonstrated the most impressive improvement.

Notably, the primary focus herein is on the improvement of Z–R relations in Mei-Yu frontal precipitation with scaled DSD models. Rainfall estimation for polarimetric radar is only briefly discussed, so more research is required to better understand the benefits of calculating polarimetric radar variables via scaled DSD. In addition, the instrumental limitations of Parsivel sensors should be further discussed. The rainfall estimations based on Parsivel^2^ DSD sensors should be validated by weather radar observations. Meanwhile, some issues might occur from research to operation such as the difference of height between Parsivel and radar. We leave that for future research with effective radar data collected.

## Figures and Tables

**Table 1:** Mei-Yu frontal precipitation events detected in three different parts of the Mei-Yu front.

Date	Regions	1-min Samples (min)	Accumulated Precipitation (mm)	Max Rain Rate (mm h^−1^)	Max Reflectivity Factor (dBZ)
2 July 2014	NJ	828	34.41	36.26	44.42
	CZ	610	9.74	26.47	41.95
	WP	454	8.60	5.74	48.22
4 July 2014	NJ	1238	95.19	86.83	53.52
	CZ	1112	107.36	71.90	49.31
	WP	104	5.91	10.54	16.19
12 July 2014	NJ	545	42.73	88.22	56.94
	CZ	584	21.18	22.33	48.26
	WP	95	10.79	48.64	52.76

**Table 2:** Sample distribution of precipitation in different rain rate classes in three parts of Mei-Yu front.

Regions	Rain Rate Classes (mm h^−1^)					
	0–10	10–20	20–30	30–40	40–50	50–60
NJ	2030	284	66	33	25	19
CZ	2073	135	45	23	15	13
WP	468	81	36	13	11	9

**Table 3:** Parameter values of the radar *Z* = *AR^b^* relations based on three different rainfall estimation methods.

Rain Type	Regions	LS		EXP		GAM	
		A	b	A	b	A	b
convective	NJ	128	1.62	238	1.81	116	1.81
	CZ	115	1.62	729	1.67	143	1.67
	WP	142	1.76	386	1.80	168	1.80
stratiform	NJ	308	1.50	1557	1.65	748	1.65
	CZ	275	1.52	1204	1.68	365	1.68
	WP	489	1.17	856	1.74	576	1.74

**Table 4:** NB and NAE errors (%) of four rainfall estimation methods are obtained via Equations (23) and (24).

Rain Type	Regions	NAE				NB			
		STD	LS	EXP	GAM	STD	LS	EXP	GAM
convective	NJ	40.1	19.1	30.3	17.9	16.6	0	−29.8	−5.4
	CZ	18.3	14.3	36.3	13.1	14.2	0	−34.3	−7.9
	WP	48.7	15.9	37.6	15.5	33.8	0	−35.7	−11.4
stratiform	NJ	52.4	33.6	31.5	24.9	43.8	0	−27.3	−18.4
	CZ	43.3	25.7	24.6	15.8	23.4	0	−21.9	−13.2
	WP	44.8	30.6	47.8	15.3	47.8	0	−33.5	−15

**Table 5:** NB and NAE errors (%) of *Z–R* (LS and GAM method), *R*(*Z_H_*, *Z_DR_*), and *R*(*K_dp_*) for precipitation in the three parts of the Mei-Yu front.

Parameters	Regions	*Z*–*R* (LS)	*Z*–*R* (GAM)	*R*(*Z_H_*, *Z_DR_*)	*R*(*K_dp_*)
NAE	NJ	27.6	25.3	9.2	17.9
	CZ	28.5	23.9	10.7	21.1
	WP	39.8	35.4	25.5	34.4
NB	NJ	0	−11.7	−3.8	−9.7
	CZ	0	−13.5	−4.9	−11.3
	WP	0	−25.4	25	32.2
